# Divergence of three *BRX* homoeologs in *Brassica rapa* and its effect on leaf morphology

**DOI:** 10.1038/s41438-021-00504-3

**Published:** 2021-04-01

**Authors:** Yuanyuan Zhang, Jianli Liang, Xu Cai, Haixu Chen, Jian Wu, Runmao Lin, Feng Cheng, Xiaowu Wang

**Affiliations:** Institute of Vegetables and Flowers, Chinese Academy of Agricultural Science, Beijing, China

**Keywords:** Polyploidy in plants, Leaf development

## Abstract

The leafy head characteristic is a special phenotype of Chinese cabbage resulting from artificial selection during domestication and breeding. *BREVIS RADIX* (*BRX*) has been suggested to control root elongation, shoot growth, and tiller angle in Arabidopsis and rice. In *Brassica rapa*, three *BrBRX* homoeologs have been identified, but only *BrBRX.1* and *BrBRX.2* were found to be under selection in leaf-heading accessions, indicating their functional diversification in leafy head formation. Here, we show that these three *BrBRX* genes belong to a plant-specific *BRX* gene family but that they have significantly diverged from other *BRX-like* members on the basis of different phylogenetic classifications, motif compositions and expression patterns. Moreover, although the expression of these three *BrBRX* genes differed, compared with *BrBRX.3*, *BrBRX.1,* and *BrBRX.2* displayed similar expression patterns. Arabidopsis mutant complementation studies showed that only *BrBRX.1* could rescue the *brx* root phenotype, whereas *BrBRX.2* and *BrBRX.3* could not. However, overexpression of each of the three *BrBRX* genes in Arabidopsis resulted in similar pleiotropic leaf phenotypes, including epinastic leaf morphology, with an increase in leaf number and leaf petiole length and a reduction in leaf angle. These leaf traits are associated with leafy head formation. Further testing of a SNP (T/C) in *BrBRX.2* confirmed that this allele in the heading accessions was strongly associated with the leaf-heading trait of *B. rapa*. Our results revealed that all three *BrBRX* genes may be involved in the leaf-heading trait, but they may have functionally diverged on the basis of their differential expression.

## Introduction

*Brassica rapa* (*B. rapa*, 2n = 20, AA) belongs to the Brassicaceae family, which includes various economically important vegetable, oilseed, condiment, and fodder crop species. *B. rapa* has a rich variety of morphotypes showing extreme traits, such as the leafy heads of Chinese cabbage, enlarged roots or stem tubers of turnips, and enlarged stems of Caixin. This phenotypic diversification is the result of artificial selection during domestication and breeding.

Previous whole-genome sequencing of *B. rapa* and its sister species *Brassica oleracea* (*B. oleracea*, 2n = 18, CC) and *Brassica nigra* (*B. nigra*, 2n = 16, BB) demonstrated that *Brassica* species experienced an extra whole-genome triplication (WGT) event after the divergence of *Arabidopsis thaliana*, followed by diploidization. Thus, the *Brassica* genomes feature three subgenomes and multiple copies of homoeologous genes. Furthermore, among the three *Brassica* subgenomes, the least fractionated subgenome (LF) is dominant over the remaining two fractionated subgenomes (MF1 and MF2) at the levels of gene density and expression^1–4^. The WGT event played an important role in the speciation and morphotype diversification of *Brassica* plants^5^.

Leafy heads are unique organs of Chinese cabbage. The leafy head trait is also observed in *B. oleracea* and *Brassica juncea* (*B. juncea*) in the forms of cabbage and heading mustard. The size, shape, uniformity, and compactness of the leafy head directly affect the attractiveness and commercial value of these vegetables. Recently, Cheng et al. investigated the genetic basis of the diversification and convergent domestication of extreme heading morphotypes of Chinese cabbage (*B. rapa*) and cabbage (*B. oleracea*)^6^. These researchers found that some orthologs of genes involved in the Arabidopsis adaxial–abaxial polarity pathway, including the *ARF3*, *ARF4*, *KAN2,* and *ATHB15* genes, were under selection in leaf-heading accessions. In addition, *BREVIS RADIX* (*BRX*) orthologs, including *BrBRX.1* (LF) and *BrBRX.2* (MF1) in *B. rapa* and *BoBRX.2* (MF1) in *B. oleracea*, were also under selection in leaf-heading accessions. These results suggested that these orthologous genes have an important role in the selection for the leaf-heading trait and that convergent subgenome parallel selection of homoeologous genes contributed to this process^6^.

*BRX*, first identified from Arabidopsis UK-1 plants with a short-root phenotype, is an important member of the *BRX* gene family, which comprises five genes: *BRX* and *BRX-LIKE1–4* (*BRXL1–4*)^7–9^. The BRX family proteins contain four highly conserved domains, including two short N-terminal domains and a tandem repeat of 55 amino acids termed BRX domains^7,8,10^. In principle, genetic redundancy generally occurs between members of the same gene family. However, it was shown that only *AtBRXL1* and no other *AtBRXL* genes could rescue *AtBRX* activity involving root growth regulation^7,8^. In contrast, nearly all monocotyledon *BRXL* genes are able to rescue the short-root phenotype of the *atbrx-*null mutant, indicating that BRX family proteins seem to be more diversified in dicotyledons than in monocotyledons^8^. The *BRX* gene is also involved in shoot growth. Arabidopsis *brx* mutants exhibit a curled-leaf phenotype and present significantly reduced cotyledon and leaf growth, whereas overexpression of *AtBRX* causes epinastic leaf growth^11^. In addition, recent research has shown that *OsBRXL4*, which is an ortholog of *AtBRX*, can also regulate rice tiller angle by affecting LAZY1 nuclear localization. However, there is currently no evidence that *AtBRX* or *AtBRXL*s also control branch angle in the dicot Arabidopsis. These results indicate that *AtBRXL*s and *OsBRXL*s have diverse functional differentiation^12^.

Although *B. rapa* and *A. thaliana* belong to the Cruciferae family, *B. rapa* has undergone additional WGT since its divergence from *A. thaliana*. Polyploidy (i.e., genome duplication) plays important roles in the divergence of duplicated gene expression and therefore in the functional divergence of duplicated genes^13–15^. In *B. rapa*, three copies of *BrBRX* have been identified, but only two copies, *BrBRX.1* and *BrBRX.2*, were found to be under selection in leaf-heading accessions, indicating that the duplicated *BrBRX* genes may be involved in leaf heading of Chinese cabbage but possibly underwent divergence in terms of either expression level or functional activity during this process. In this study, we investigated the consequence of polyploidy on *BrBRX* structure, evolution, expression, and function. Our results highlight the role of *BrBRX* homoeologs in leaf morphology regulation, and our findings demonstrate both the expression and functional divergence of duplicated *BrBRX* genes in *B. rapa*. This work provides insights into our understanding of the role of *BrBRX* genes in the regulation of leaf morphology, especially in the formation of the leafy head trait.

## Results

### Three *BrBRX* genes belong to a highly conserved plant-specific *BRX* gene family

**Fig. 1:**
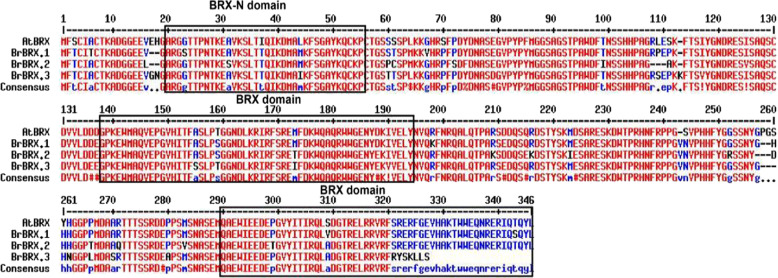
Multiple alignments of three BrBRX and AtBRX protein sequences. Fully and partially conserved residues (present in >50% of the aligned sequences) are highlighted in red and blue, respectively. Three conserved domains, one BRX-N domain and two BRX domains, are indicated in boxes

In *B. rapa*, three *BrBRX* homoeologs produced by WGT and distributed across different chromosomes have been annotated in the BRAD (http://brassicadb.org): *BrBRX.1* (BraA09g033250.3 C/Bra023219, A09), *BrBRX.2* (BraA08g009040.3 C/Bra035521, A08), and *BrBRX.3* (BraA05g023900.3 C/Bra033869, A05). The open reading frames of the three *BrBRX* genes varied in length from 975 to 1026 bp, encoding proteins of 341, 337, and 324 aa, respectively. *BrBRX.1* and *BrBRX.2* contain five exons, whereas *BrBRX.3* contains four exons (Table S1). We isolated the three *BrBRX* genes from *B. rapa* accession Chiifu-401/42 based on their sequences in the BRAD^16^. The three predicted BrBRX proteins were 87–91% identical, sharing high similarity (88–93%) with the sequence of Arabidopsis BRX (AtBRX) (Table S2). Sequence analysis of the three predicted BrBRX proteins revealed that each contained three highly conserved domains: one domain located at the N-terminus (BRX-N) and two so-called BRX domains in the C-terminal region (Fig. 1). These are the common characteristic features of BRX family proteins. As previously reported, at the N-terminus of most BRX family proteins, there are two short conserved domains comprising approximately 10 and 25 amino acids. The N-terminus has a minor role, but the two BRX domains are of critical importance for BRX function. Here, we investigated only one N-terminal domain because the domain with 10 amino acids was not annotated by the NCBI Batch CD-search tools (https://www.ncbi.nlm.nih.gov/Structure/bwrpsb/bwrpsb.cgi). Moreover, although all three deduced BrBRX proteins contain the three conserved domains, in the second BRX domain region, the BrBRX.3 protein shows greater structural divergence than does BrBRX.1 and BrBRX.2. This might have consequences for the functional diversification of the proteins.

In Arabidopsis, the highly conserved *BRX* gene family consists of five members: *BRX* and *BRXL1–4*. Based on the sequences of the three *BrBRX* genes and the results of the homology searches, in *B. rapa*, four additional *BRX* family members, *BRXL1* to *4*, including 10 BRXLs with three conserved domains, were identified (Table S3). In theory, each *BRX* family gene of Arabidopsis should have three copies in *B. rapa*, since this species experienced a WGT event after its divergence from Arabidopsis. However, only the *AtBRX* gene had three copies, while the remaining *BRXL* members had one or two copies in the *B. rapa* genome, suggesting that most *BRX* family genes experienced different degrees of gene loss after the WGT event.

**Fig. 2:**
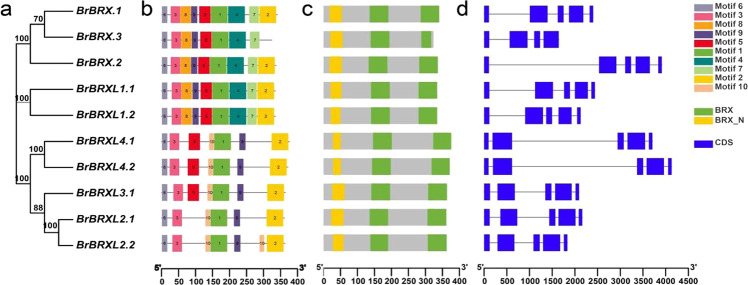
Phylogenetic relationships, conserved domains, conserved motifs, and structure of BRXL genes from B. rapa. **a** The phylogenetic tree was constructed based on the full-length proteins of BRXLs using MEGA 5 software (www.megasoftware.net). **b** Conserved domain location of BRXL proteins. The BRX-N domain and two BRX domains are indicated by yellow and green boxes, respectively. **c** The motif distribution in BRXL proteins was predicted using the MEME web server. The motifs, numbered 1–10, are displayed in different colored boxes. **d** Exton–intron structure of *BRXL* genes. The blue boxes indicate exons, and the gray lines indicate introns

Although BRXLs of *B. rapa* are characterized by three conserved domains and similar gene structures (Fig. 2a–d), only three BrBRXs and two BrBRXL1s shared similar conserved motif compositions with specific motifs 4, 7, and 8, which may provide unique functional specificity compared with those of other BRX family proteins (Fig. 2b). To explore and deepen our understanding of the evolutionary origin of these three *BrBRX* genes, we first identified all BRX family protein sequences in the 21 sequenced Brassicaceae species (Table S4) and then constructed a phylogenetic tree (Fig. S1). *B*RX family genes had apparently already diverged before the separation of gymnosperms and angiosperms, and they were divided into three clades: *BRX/BRXL1*, *BRXL2/BRXL3,* and *BRXL4*. Further evolutionary analysis showed that *AtBRX* and *AtBRXL1* were in fact paralogous gene pairs that originated from the α WGD event, and AtBRXL2 and AtBRXL3 were also produced from the α duplication event^17^. Since *B. rapa* shares the same evolutionary history with Arabidopsis but has undergone an additional WGT event, in *B. rapa*, the three *BrBRX* genes and two *BrBRXL1* genes must have originally been produced from the α duplication event, and they may have similar or identical functions but be functionally divergent from other BRXLs of different clades. These results are consistent with previous results in which only *AtBRXL1* and no other *BRXL* genes when constitutively expressed could rescue AtBRX activity involving root growth regulation in Arabidopsis. However, the activity of *AtBRXL1* is not redundant with that of *AtBRX* in vivo, presumably because, compared with *AtBRX*, the former is differentially expressed^7^.

### Three BrBRX genes exhibit distinct expression patterns

Genome polyploidy has considerable effects on differences in expression between duplicate genes, including spatiotemporal silencing and upregulated or downregulated expression of one of the duplicated genes^14,15,18^. This might have happened in the *BRX* gene family of *B. rapa*. Here, we investigated the expression of *BrBRX* and *BrBRXL1* in five different tissues (root, stem, leaf, flower, and silique tissues) of *B. rapa* using the RNA-sequencing data described by Tong et al.^19^. Although three *BrBRX* genes and two *BrBRXL1* genes from the same phylogenetic clade exhibited low expression across all detected samples, their expression patterns had different characteristics (Table S5 and Fig. S2). *BRXL1.2* expression was nearly absent in the five different tissues, while *BRXL1.1* expression was predominant in the roots. In addition, the three *BrBRX* genes showed different expression patterns. *BrBRX.1* and *BrBRX.3* exhibited relatively high expression in the roots compared with the other tissues, and *BrBRX.2* exhibited higher expression in the stems than in other tissues. In the leaves, *BrBRX.1* and *BrBRX.2* exhibited higher expression than did *BrBRX.3*.

**Fig. 3:**
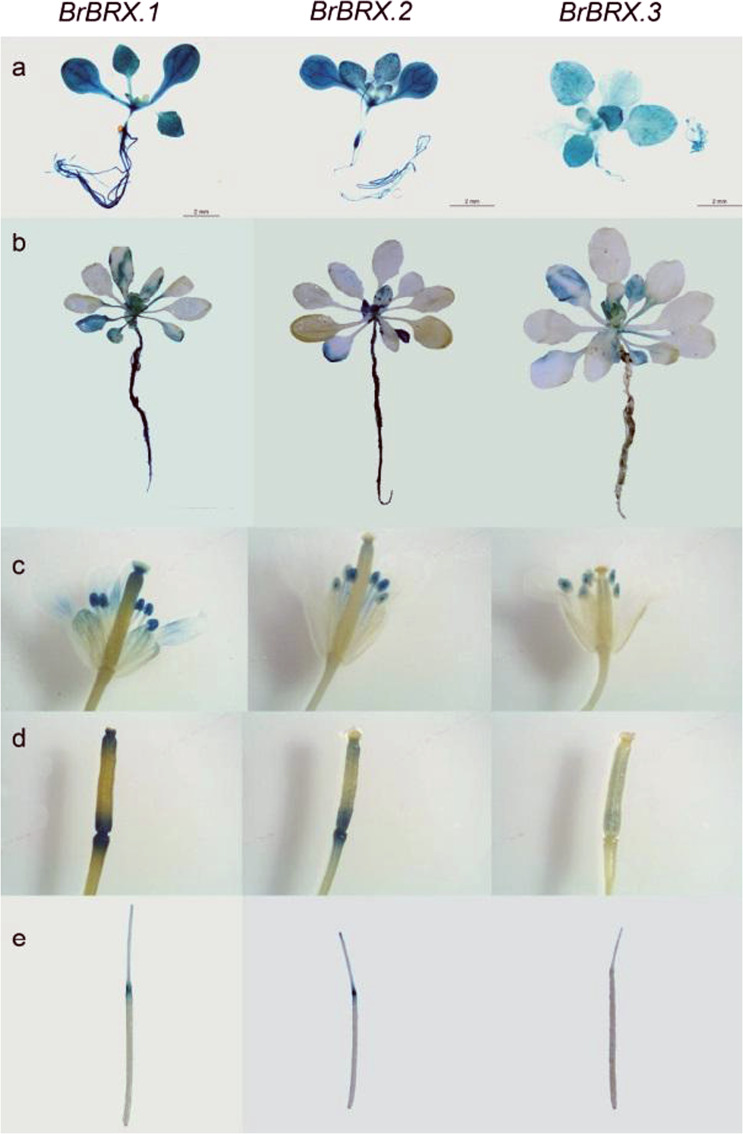
Histochemical GUS staining of ProBrBRX::GUS transgenic Arabidopsis lines at different developmental stages. **a** Three-week-old seedlings. **b** Five-week-old whole plants. **c** Flowers. **d** Immature silique. **e** Mature silique. Plants at stage (**a**) were grown on MS media, and plants at the other stages were grown in soil

To better understand the expression of the three duplicated *BrBRX* genes, we generated transgenic plants harboring a GUS reporter driven by each *BrBRX* promoter (Pro _*BrBRX*_:GUS) to analyze *BrBRX* expression. The reporter gene expression in the transgenic Arabidopsis lines revealed that *BrBRX.1* and *BrBRX.2* showed similar and relatively high expression patterns, while *BrBRX.3* showed distinct expression patterns during different development stages of *Arabidopsis thaliana* (Fig. 3a–e). Histochemical analysis revealed that the Pro _*BrBRX.1*_:GUS and Pro _*BrBRX.2*_:GUS transgenic lines had higher GUS activity in the seedlings, mature roots, flowers, and junctions between seed pods and seed stalks than did the Pro _*BrBRX.3*_:GUS lines. However, GUS expression was higher in the seedling roots and flower structures, including the veins of petals, stigmas, and anthers, of the Pro _*BrBRX.1*_:GUS lines than in the Pro _*BrBRX.2*_:GUS lines, in which GUS expression in the stigmas and petal veins was undetectable. In the Pro _*BrBRX.3*_:GUS transgenic lines, prominent GUS staining was detected in the seedling leaves, roots, and anthers, while no GUS staining was detected in mature roots, petals, stigmas, or siliques (Fig. 3a–e). The GUS histochemical data again confirmed that the three *BrBRX* genes have undergone divergence in terms of expression.

To further explore the potential functions of these three *BrBRX* genes and the regulation of their expression, their promoter sequences (a 2000 bp DNA fragment upstream of the ATG start codon) were analyzed. According to the results, multiple cis-acting elements associated with responses to plant hormones, such as auxin, methyl jasmonate (MeJA), gibberellins (GAs), abscisic acid (ABA), and salicylic acid (SA), were found. In addition, some light-responsive cis-acting elements were also found (Fig. S3). Moreover, there were differences in cis-regulatory elements among the three *BrBRX* promoters.

**Fig. 4:**
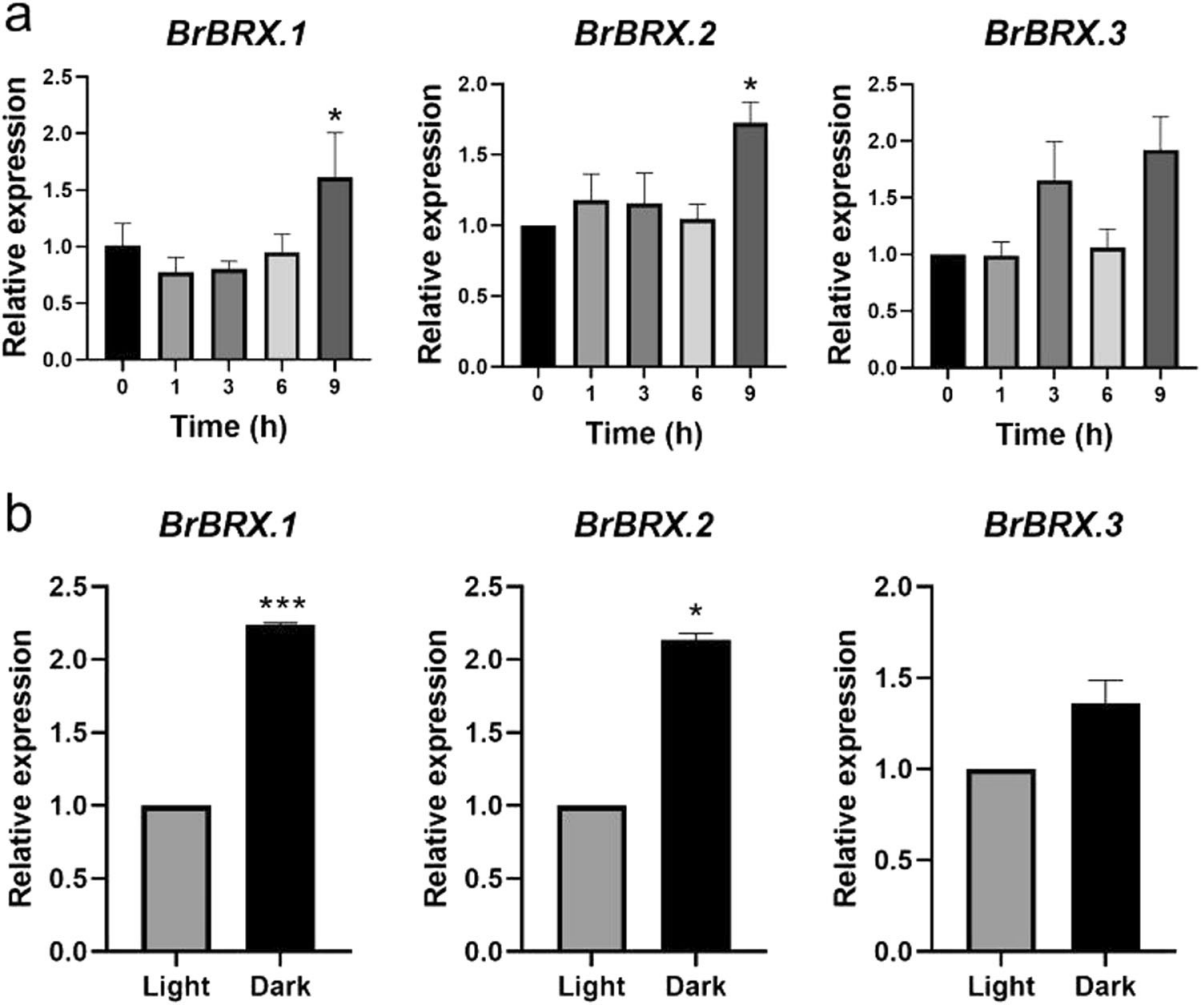
**The expression patterns of three BrBRX genes under IAA or light/dark treatments.** The expression of BrBRX genes under IAA (**a**) or light/dark (**b**) treatments was determined by q-RT-PCR analysis. The data were normalized to the expression level of *BrActin2*. The error bars represent the standard deviations of three independent biological repeats. The asterisks represent significant differences via *T*-tests (*P ≤ 0.05; **P ≤ 0.01; and ***P ≤ 0.001)

It is known that *BRX*s are involved in auxin signaling. To determine the transcript levels of the three *BrBRX* genes in response to auxin, real-time PCR was performed to determine the responsiveness of the three *BrBRX* genes to auxin. With IAA treatment, the expression levels of *BrBRX.1* and *BrBRX.2* increased by a factor of two at 9 h compared to 0 h (Fig. 4a). We also investigated the expression levels of the three *BrBRX* genes in response to light/dark treatment. Our results showed that the expression of *BrBRX.1* was strongly induced and that *BrBRX.2* was slightly induced after dark treatment (Fig. 4b). *BrBRX.1* and *BrBRX.2* were responsive to both the IAA and dark treatments, while *BrBRX.3* was not responsive to either of these treatments. Taken together, these results suggested that the three *BrBRX* genes in *B. rapa* may be involved in auxin and light signaling but that they respond differently to the treatments.

### Three *BrBRX* homoeologs exhibit functional diversification

**Fig. 5:**
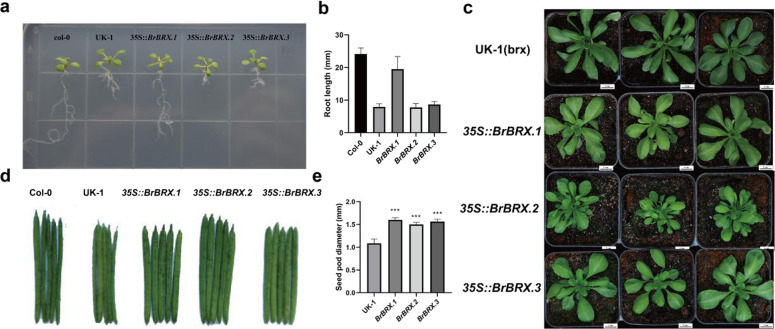
Complementary phenotype of the Arabidopsis natural mutant UK-1 transformed with three BrBRX genes. **a** Root phenotypes of three *BrBRX* transgenic Arabidopsis seedlings at two weeks. **b** Lengths of primary roots of two-week-old seedlings. **c** Rosette phenotypes of *BrBRX* transgenic Arabidopsis seedlings at four weeks. **d** Silique phenotype of *BrBRX* transgenic Arabidopsis seedlings. **e** Diameter of siliques of *BrBRX* transgenic Arabidopsis

To determine whether the three *BrBRX* genes are functionally equivalent to *AtBRX*, we performed transgenic complementation of the Arabidopsis *brx* mutant short-root phenotype through ectopic overexpression of *BrBRX* genes via the cauliflower mosaic virus 35 S promoter. *BrBRX.1* rescued *brx* root growth to wild-type levels, whereas *BrBRX.2* and *BrBRX.3* did not (Fig. 5a, b). Interestingly, we observed a significant difference in leaf phenotype between the control lines and lines complemented by *BrBRX.2* (Fig. 5c). The rosette leaf number significantly increased and the rosette leaf area was significantly reduced in the *brx* lines complemented by the *35* *S::BrBRX.2* transgene compared with the *brx* control lines. However, such differences in leaf phenotype were not observed between the control line and lines complemented by *35* *S::BrBRX.1* or *35* *S::BrBRX.3*. In addition, we also observed that the silique diameter increased significantly in the lines complemented by these three *BrBRX* genes compared with the *brx* control line (Fig. 5d–e). These results suggested that the three *BrBRX* genes have functional differences in root elongation regulation and that *BrBRX* genes may have roles in the regulation of leaf morphology and silique development.

### Three *BrBRX* homoeologs control leaf morphology

**Fig. 6:**
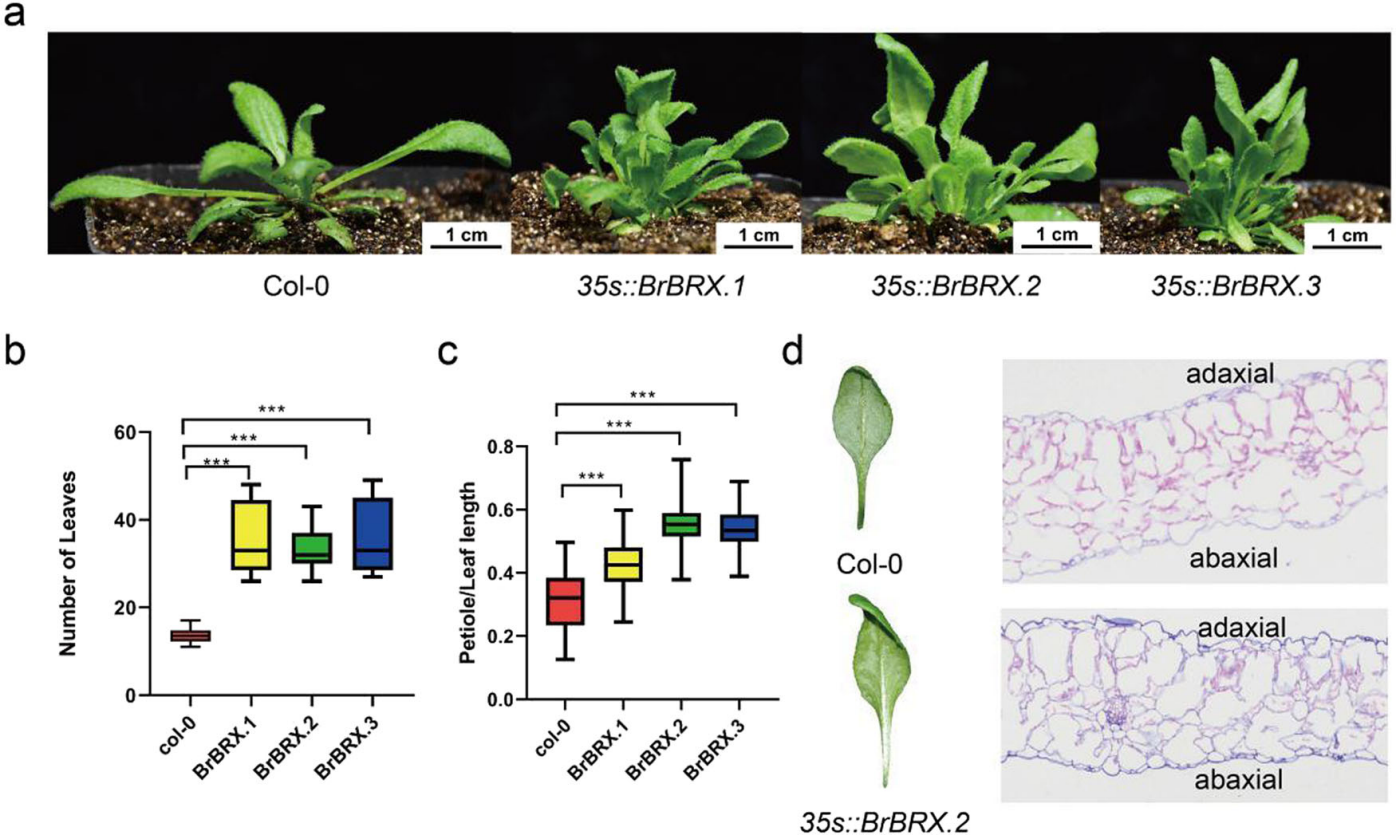
Leaf phenotypic variation of BrBRX transgenic Arabidopsis. **a** Leaf phenotypes of Col-0 and *BrBRX* transgenic plants. **b** Leaf number of *35* *s*::*BrBRX* and Col-0. **c** Petiole ratio of *35* *s*::*BrBRX* and Col-0. **d** Cell structure of leaves of *35* *s*::*BrBRX.2* and Col-0

To test whether the three *BrBRX* genes are involved in the regulation of leaf morphology, the full-length cDNA of each *BrBRX* under the control of the CaMV 35 S promoter was transformed into Arabidopsis Col-0. A significant difference in leaf phenotype was observed, confirming previous observations (Fig. 6a). Compared with the control lines, each gain-of-function line overexpressing one of the three *BrBRX* genes exhibited similar pleiotropic leaf phenotypes. The rosette leaf number significantly increased (Fig. 6b), and the rosette leaf area was significantly reduced. In addition, the leaf petiole length tended to increase, which was confirmed by measuring the petiole length/leaf length ratio, which significantly increased (Fig. 6c). Interestingly, we also observed leaf epinastic growth, i.e., downward leaf curling and a significant reduction in leaf angle. However, the epinastic leaf growth was not always significant, possibly due to the inherent plasticity of leaf development. These results demonstrated that all three *BrBRX* genes were involved in the regulation of leaf morphology.

We further characterized the anatomical features of the curling leaves of *35* *S::BrBRX.2*. Wild-type leaves consisted of a palisade parenchyma layer on the adaxial side and a spongy parenchyma layer on the abaxial side. The cells of the palisade layer were tightly packed, whereas the spongy parenchyma was more loosely arranged (Fig. 6d). However, in *35* *S::BrBRX.2* curling leaves, the palisade parenchyma layer was absent; the spongy parenchyma cells were packed on the adaxial side as well as on the abaxial side (Fig. 6e). These results suggest that *35* *S::BrBRX.2* might cause changes in leaf adaxial–abaxial patterning.

### BrBRX under selection and with dominant expression is associated with leafy head formation

Previous studies have suggested that *BrBRX.1* and *BrBRX.2* were under strong selection in leaf-heading *B. rapa*^6^. To further validate the selection signals for the heading morphotype, we first estimated the level of the genetic variation of *BrBRX.1* and *BrBRX.2* by examining our resequencing dataset, which consisted of 199 *B. rapa* accessions: 47 were Chinese cabbage heading *B. rapa* (H-Br) accessions, whereas the other 155 were nonheading *B. rapa* (NH-Br) accessions. A C/T mutation (located at position 1698 in the CDS) in *BrBRX.1* and a T/C mutation (located at position 1318 in the CDS) in *BrBRX.2* were identified, which indicated a significantly biased distribution between the H-Br and NH-Br groups (Table S6). We then developed one KASP marker for each gene to genotype in an extended germplasm collection consisting of 908 *B. rapa* accessions, which included 199 heading and 709 nonheading cultivars (176 pak choi, 134 Caixin, 109 turnip, 113 sarson oil and 177 others). For the C/T SNP in *BrBRX.1*, 80.4% of the heading accessions were of the C genotype, while only 12.1% of the accessions were of the T genotype (Fig. S4a). In contrast, in the nonheading group, 64.5% of the accessions were of the T genotype, whereas 24.6% were of the C genotype. For the T/C mutation in *BrBRX.2*, a high percentage (88.2%) of heading accessions were of the T genotype (Fig. S4b). In contrast, 62% of the nonheading accessions were of the C genotype, while 27% of the accessions were of the T genotype. Taken together, the results suggested that both the C/T mutation in *BrBRX.1* and the T/C mutation in *BrBRX.2* clearly exhibited biased distribution between the H-Br and NH-Br groups and that the H-Br alleles of *BrBRX.1* (*P* = 8.43E−48; Table S7) and *BrBRX.2* (*P* = 6.51E−49; Table S7) were strongly associated with the leaf-heading trait.

To further investigate whether *BrBRX.1* and *BrBRX.2* are expressed dominantly instead of the nonselected homoeolog *BrBRX.3* during leafy head formation of Chinese cabbage, we investigated the expression pattern of the three *BrBRX* genes at the rosette stage (week 4) and heading stage (week 18) in the heading cultivar Chiifu and at identical time points (week 4 and week 18) in the nonheading accession Taicai via transcriptome profile sequencing. Taicai has a no-heading stage and stays in the rosette stage after the seedling stage. In the heading cultivar Chiifu, the expression of three *BrBRX* genes was relatively low at the rosette stage, while at the heading stage, the expression of *BrBRX.1* and *BrBRX.2* significantly increased compared with that of the nonselected homoeolog *BrBRX.3*. In contrast, in the nonheading accession Taicai, the transcript levels of all three *BrBRX* genes were relatively low and did not change significantly at either the rosette or corresponding heading stage (Fig. S5).

**Fig. 7:**
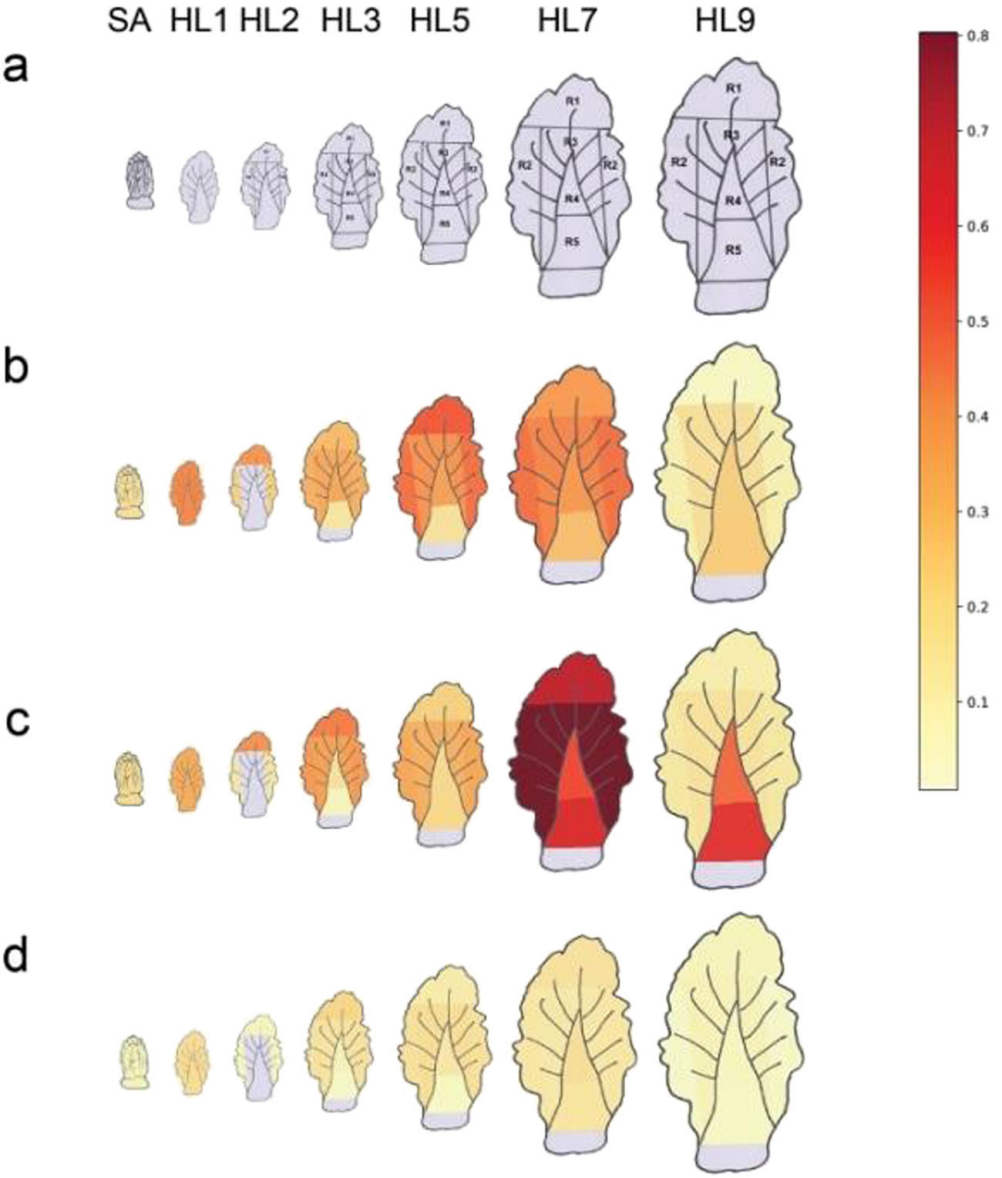
Expression mapping of BrBRX genes in leafy heads of Chinese cabbage. **a** Schematic diagram showing how each sample was taken. **b**–**d** The images of *BrBRX.1*, *BrBRX.2,* and *BrBRX.3* expression, respectively. The pictograms were generated on the basis of RNA-seq data (from left to right): SA, head leaves <2 cm long, with a shoot apical meristem; HL1, head leaf 1; and HL2, 3, 5, 7, and 9, head leaves 2, 3, 5, 7, and 9, respectively. Different regions of the head leaf are labeled using light gray lines

We also performed transcriptome profile sequencing of different head leaves at the heading stage. The head leaves were wrinkled, with an upward curvature, and have broad petioles, and they differ in age, size, and degree of incurvature from the inner central leaves to the outer head leaves. Moreover, different regions on the same head leaf showed different degrees of incurvature. Thus, several different regions of different head leaves were sampled to perform the transcriptome analysis (Fig. 7a). Our results showed that the expression of *BrBRX.3* was very low in all the measured samples, including the SA and all HL samples, and compared with *BrBRX.3*, *BrBRX.1* and *BrBRX.2* were significantly overexpressed (Fig. 7b–d and Table S8). In addition, *BrBRX.1* and *BrBRX.2* displayed similar or overlapping expression patterns in different regions of different head leaves, with relatively high expression on the top and margin regions of the head leaves, except for external HL9. Interestingly, *BrBRX.1* and *BrBRX.2* exhibited higher expression levels across five different regions of HL7 compared with other HLs and SAs. Moreover, in external HL9, *BrBRX.1* and *BrBRX.2* exhibited very low expression levels but exhibited higher expression in the petioles than in leaf blade regions. These results showed that the leaf-heading selection genes *BrBRX.1* and *BrBRX.2* had stronger dominant expression than did their nonselected homoeolog, *BrBRX.3*, suggesting that strong functional differentiation of the three *BrBRX* homoeologs occurred after selection for leaf heading.

## Discussion

### Divergence and origin of *BrBRX* genes

Comprehensive sequence analysis is the first step in the functional dissection of genes of interest or gene families. In this work, based on sequence analysis, we found that three *BrBRX* homoeologs in *B. rapa* formed from a WGT event shared high similarity (88–93%) with Arabidopsis *BRX*. *BRX*s belong to a plant-specific *BRX* gene family comprising five members. Our phylogenetic analyses suggested that the *BRX* gene family evolved before the divergence of gymnosperms and angiosperms and expanded after the divergence of monocotyledons and dicotyledons.

Previous studies have suggested that *BRX* family genes are more diversified in dicotyledons than in monocotyledons. This has been suggested because, in Arabidopsis, only *AtBRX* and *AtBRXL1* could complement *brx-*null mutants when these genes were constitutively expressed, while nearly all monocotyledon BRXLs could complement *brx*-null mutants^8^. In the 21 Brassicaceae species studied here, the *BRX* gene family could be divided into three distinct clades, and members of the different clades were characterized by their specific motif composition, which aligned with the differences in the molecular functions among the members from three clades. In fact, it has been demonstrated via genetic analyses in Arabidopsis that only the members of clade I, *BRX* and *BRXL1*, have equivalent activity involving root growth^7,8^. Our study also found that *AtBRX* and *AtBRXL1* constitute a duplicated gene pair produced by the α WGD event, and the same was true for *AtBRXL2* and *AtBRXL3*. In principle, gene duplication creates functionally identical copies that should be fully or partially redundant. This could explain why *BRX* and *BRXL1* have equivalent activity but functionally differ from members of other clades. *B. rapa* has the same evolutionary history as Arabidopsis but has undergone an additional WGT event. Therefore, our phylogenetic classification and conserved motif composition analyses suggested that the three BrBRXs and two BrBRXL1 sharing high amino acid identities with AtBRX-AtBRXL1 might have similar functions in *B. rapa* but have functionally diversified from other *BrBRXL* genes.

*B. oleracea*, a sister species of *B. rapa*, also contains three *BRX* genes whose evolutionary history is similar to that of *B. rapa*, indicating that *BRX* genes in *B. oleracea* and *B. rapa* might have similar functions. In fact, our previous studies showed that *BrBRX.1* and *BrBRX.2* in *B. rapa* and *BoBRX.2* in *B. oleracea* were under selection in leaf-heading accessions, indicating that these genes play roles in the leaf-heading trait. Therefore, our study of the evolution and function of *BRX* genes in *B. rapa* might help to elucidate the functional activity of BRX proteins in *B. oleracea* and other *Brassica* species.

### Expression divergence of *BrBRX* genes in *B. rapa*

As previously reported, both *AtBRX* and *AtBRXL1* have equivalent activities in root growth. However, there is a lack of redundancy between them in vivo. This suggested that *AtBRX* and *AtBRXL1* might have diverged due to differences in expression rather than differences in protein activity. Moreover, expression analyses revealed that *AtBRX* is expressed at a level 9 to 10 times higher than *AtBRXL1* is in the roots, although both genes are weakly expressed^7^. In the current study, we found that the three *BrBRX* genes and two *BrBRXL1* genes also showed different expression patterns in five different tissues of *B. rapa* (Fig. S1). In addition, all three *BrBRX* homoeologs were expressed at very low levels in the five different tissues, but the expression profiles of the three *BrBRX* homoeologs differed. Histochemical analysis of GUS activity confirmed that, compared with *BrBRX.3*, *BrBRX.1* and *BrBRX.2* showed similar expression patterns during the different developmental stages of Arabidopsis. qRT-PCR experiments also revealed that, compared with *BrBRX.3*, *BrBRX.1* and *BrBRX.2* had similar responses to auxin and light/dark treatment in *B. rapa*. Moreover, our results showed that, compared with the nonselected *BrBRX.3* gene, the selected *BrBRX.1* and *BrBRX.2* genes exhibited strong, dominant expression in all the examined head leaves at the heading stage of Chinese cabbage, and *BrBRX.1* and *BrBRX.2* exhibited similar expression patterns, with higher expression in the outer head leaves (HL7) than in the other leaves.

Previous studies have shown that the difference in expression of polyploidy-derived duplicated genes is one of the most important indicators of subfunctionalization and neofunctionalization of duplicated genes^13–15^. Our results confirmed that, compared with the two *BrBRXL1* genes, the three *BrBRX* homoeologs exhibit different expression patterns and expression levels, indicating strong functional divergence between the three *BrBRX* homoeologs and two *BrBRXL1* homoeologs.

### Functional divergence of three *BrBRX* genes in *B. rapa*

The role of *BRX* genes in root growth has been well studied. However, a role for *BRX*s in shoot growth has not been reported because of the remarkable plasticity of leaves during development. After careful analysis, Beuchat et al. revealed that *BRX* is also involved in shoot growth. In Arabidopsis, a *BRX* loss-of-function mutation significantly affected rosette leaf area and dry rosette weight but did not affect leaf number. However, overexpression of *BRX* in Arabidopsis resulted in epinastic leaf growth^11^. More recently, *OsBRXL4*, an ortholog of *AtBRX*, was demonstrated to regulate rice tiller angle by affecting LAZY1 nuclear localization ^12^.

In our study, the functional contributions of the three *BrBRX* genes to root growth were assessed in the Arabidopsis *brx* mutant (UK-1) with a short-root phenotype by complementation tests. *BrBRX.1* rescued *brx* root growth, whereas *BrBRX.2* and *BrBRX.3* did not. Unexpectedly, all three *BrBRX* genes resulted in significantly increased silique diameter relative to that of the *brx* mutant, and *BrBRX.2* affected the rosette leaf number and leaf area of the *brx* mutant. These phenotypes appeared to be caused by very high levels of transgene expression during the mutant complementation analysis. We thus tested the role of the three *BrBRX* genes in leaf morphology by overexpressing each of the three *BrBRX* homoeologs in Arabidopsis Col-0. The transgenic gain-of-function lines of each homoeolog exhibited similar pleiotropic leaf phenotypes, which included an increased rosette leaf number, a reduced rosette leaf area, and an increased petiole length/blade length ratio, with leaf epinastic growth and decreased leaf angle (upward growth). However, leaf epinastic growth was not always severe, possibly due to the remarkable plasticity of leaf development, in which environmental factors often play an important role. Our results provide genetic evidence that all three *BrBRX* genes are involved in the regulation of leaf morphology and that they may have functional differences in terms of root growth. However, there is no evidence that *BRX*s or *BRXL*s in Arabidopsis and rice also control leaf number, petiole length, or silique size.

In Arabidopsis, gain-of-function lines overexpressing full-length BRX proteins or the C-terminal fragment displayed leaf epinasty. Beuchat et al. observed that the size of epidermal cells of plants overexpressing the C-terminus of AtBRX increased compared with that of the wild-type control plants; the researchers suggested that leaf epinasty is attributed to excessive cell expansion rather than a patterning defect^11^. However, our results demonstrated that the leaf adaxial–abaxial patterning was altered in plants overexpressing full-length *BrBRX.2*, suggesting that leaf epinasty possibly occurred due to an adaxial–abaxial patterning defect. Moreover, previous studies of leaf adaxial–abaxial patterning mutants found that the plants generally presented a leaf curling phenotype^20^. Thus, our finding is a first hint of the function of the *BRX* gene in leaf adaxial–abaxial pattern regulation, and the results indicate that *BrBRX* in *B. rapa* and *AtBRX* in Arabidopsis may have different functions in leaf number and leaf adaxial–abaxial pattern regulation, although this remains to be determined.

### *BrBRX* genes are crucial candidates for leafy heading in Chinese cabbage

Compared to other nonheading *B. rapa* plants, Chinese cabbage has a uniform, compact leafy head composed of several enlarged, winged, crinkled, upwardly incurved leaves^21–23^. Upward leaf curvature is an essential prerequisite for leafy head formation^24^. Upward leaf growth is commonly attributed to a reduction in leaf angle, while leaf incurvature is attributed to a change in leaf adaxial–abaxial patterning^20^ or an imbalance in the cell expansion rate between the adaxial and abaxial surfaces of a leaf^25^.

Loss- or gain-of-function adaxial–abaxial pathway genes generally result in an upward-curling or downward-curling leaf phenotype due to alterations in the adaxial–abaxial patterning system^20,26^. The *HYL1* gene is responsible for the leaf abaxial determinant miR166^27,28^, and Arabidopsis *hyl1* mutants display an inward-curling leaf phenotype^29^. *BcpLH*, a homolog of the Arabidopsis *HYL1* gene, was isolated from Chinese cabbage and demonstrated to affect the inward curvature of folding leaves of Chinese cabbage^30^. Moreover, our previous study found that some orthologs of Arabidopsis adaxial–abaxial polarity pathway genes, such as *ARF3*, *ARF4*, *KAN2,* and *ATHB15*, are under selection in leaf-heading accessions^6,31^, suggesting that leaf adaxial–abaxial patterning could be involved in head leaf incurvature and subsequent leafy head formation.

*BrBRX.1* and *BrBRX.2* in *B. rapa* are also under selection in leaf-heading accessions^6^. In this study, we provided genetic evidence that all three *BrBRX* genes could control leaf morphology, especially by regulating leaf curvature and leaf angle. We also revealed that the leaf epinastic phenotype regulated by *BrBRX*s may be caused by alterations in leaf adaxial–abaxial patterning, although the detailed mechanism remains unclear. Moreover, we genotyped a C/T mutation in *BrBRX.1* and a T/C mutation in *BrBRX.2* showing biased distribution between heading and nonheading accessions and then confirmed that the alleles of the heading accessions were strongly associated with the leaf-heading trait in a large germplasm collection of 999 *B. rapa* accessions, and compared with nonselected *BrBRX.3*, *BrBRX.1,* and *BrBRX.2* were dominantly expressed relative at the heading stage of Chinese cabbage. These results further demonstrated that selection of *BrBRX.1* and *BrBRX*.2 could be involved in leafy head formation.

Many factors, such as temperature, light, and auxin concentration, are closely related to leafy head formation. Previous studies have demonstrated not only that exogenous application of auxin influences the formation of leafy heads but also that the relatively high content and uneven distribution of endogenous auxin in Chinese cabbage can improve the characteristics of head formation, as reviewed by He et al.^23^, suggesting that auxin concentration and its uneven distribution are associated with leafy head formation. In this study, when the promoter region sequences ~2 kb upstream of the three *BrBRX* genes were analyzed, several cis-acting elements responsive to plant hormones including auxin, ABA, and GAs were observed. In addition, some light-responsive cis-acting elements were found. We thus confirmed that the expression of *BrBRX.1* and *BrBRX.2* was upregulated in response to auxin and light/dark treatment, while the expression of nonselected *BrBRX.3* was not. The results implied that *BrBRX* may be involved in leafy head formation by mediating auxin and light signaling.

In addition, Arabidopsis mutant studies have indicated that *AtBRX* may act in root growth through a cross-talk network involving brassinolide (BR), auxin, ABA, or cytokinin^32–36^. It is well known that phytohormones such auxin and BR play critical roles in modulating plant architecture. Thus, it will be intriguing to test whether *BrBRX*s are also involved in cross-talk between auxin and BR signaling pathways with respect to leafy head formation in Chinese cabbage.

Taken together, our results provided additional evidence that three *BrBRX* homoeologs experienced strong functional divergence after selection. *BrBRX.1* and *BrBRX.2* under selection may be candidate genes responsible for leafy head formation in Chinese cabbage, and their functional characterization should be investigated in future studies.

## Materials and methods

### Plant growth, auxin, and light/dark treatment

*B. rapa* cultivar Chiifu was germinated and grown in the field until leafy heads formed at the Chinese Academy of Agricultural Sciences (Beijing, China) during the autumn of 2017. The plants were used for the cloning of *BrBRX* genes and expression pattern analysis of those genes at the heading stage.

For auxin and light/dark treatments, Chiifu-401/42 seeds were grown on Murashige and Skoog (MS) media in a culture room at 25 °C under a 16 h light/8 h dark photoperiod. Five-week-old seedlings with 4–5 true leaves were transferred to a growth chamber for three days to acclimate before treatments. For the light/dark treatment, the seedlings were divided into two groups and placed light and darkness for 48 h each, after which seedling leaves were sampled. For auxin treatment, whole seedlings were transplanted into 1/2-strength liquid MS media with 100 mM IAA (indole-3-acetic acid), and seedling leaves were sampled at 1, 3, 6, and 9 h; untreated seedlings were used as controls at the same time points. All the samples were quickly frozen in liquid nitrogen and stored at −80 °C for further qRT-PCR analysis.

*Arabidopsis thaliana* ecotypes Col-0 and UK-1 (N6879) obtained from the Nottingham Arabidopsis Stock Centre were grown at 22 °C under a 16/8 h photoperiod for functional analysis.

### Sampling and transcriptome sequencing

To investigate the expression patterns of *BrBRX*s at the rosette stage (week 4) and heading stage (week 18) of heading accession Chiifu and at the same time points in the nonheading accession Taicai, young central leaves (~2 cm long) were collected from Chiifu and Taicai plants during development. Two biological repeats were used for RNA isolation.

To determine the expression patterns of the *BrBRX* genes at the heading stage of Chinese cabbage, three individual mature leafy heads of Chiifu were harvested. All head leaves (leaf length >2 cm) from the outer leaves to inner leaves were separated and collected sequentially. The young central leaves (leaf length < 2 cm) with remaining inner small leaves and shoot apical meristem together were not separated and were collected as a composite sample, referred to as ‘SA’. A total of 30 head leaves (leaf length > 2 cm) and an SA sample were obtained from a leafy head. From the inside to the outside, every three head leaves with similar size and similar morphology were classified into a group, and then one leaf was selected from each group. As a result, 10 representative head leaves were selected, designated HL1-HL10. Among all the collected samples, the SA, HL1, HL2, HL3, HL5, HL7, and HL9 samples were ultimately selected for transcriptome analysis. Moreover, for HL3, HL5, HL7, and HL9, five regions, the top (R1), outer margin (R2) and middle region (R3) of the blade and the top (R4) and middle (R5) regions of the petiole, were sampled. For HL2, only the blade and petiole were sampled (separately). A total of 48 samples from two biological replicates (two leafy heads) were ultimately used for transcriptome sequencing via the Illumina platform.

### Identification and phylogenetic analysis of *BRX* family genes

Gene and genome datasets for 14 species of Brassicaceae were downloaded from the BRAD (http://brassicadb.org/brad/): *B. rapa* (v1.5), *B. oleracea* (v1.1), *B. nigra* (v1.1), *B. juncea* (v1.5), *B. napus* (v5.0), *R. sativus* (v1.0), *A. arabicum*, *A. lyrata*, *C. sativa* (v2.0), *C. rubella*, *L. alabamica*, *T. halophila*, *S. parvula* and *S. irio*. The genomic dataset for *D. sophia* was downloaded from the Joint Genome Initiative database (https://phytozome.jgi.doe.gov/pz/portal.html), and the *B. retrofracta* and *C. hirsuta* datasets were obtained from Kliver et al. (2018)^37^ and Gan et al. (2016), respectively^38^. The *C. himalaica* and *T. arvense* genomic datasets were obtained from Zhang et al. (2019)^39^ and Dorn et al. (2015), respectively^40^, and the genomic information of *A. thaliana* (https://www.arabidopsis.org/index.jsp), *Oryza sativa* (https://rapdb.dna.affrc.go.jp/), *Populus* (https://phytozome.jgi.doe.gov/pz/portal. html#), *Amborella trichopoda* (https://phytozome.jgi.doe.gov/pz/portal.html#), and *Ginkgo biloba* (http://gigadb.org/dataset/100613) was also downloaded.

The hidden Markov model (HMM) profiles of the BRX_N domain (Pfam:13713) and BRX domain (Pfam:08381) were used to search all BRX family protein sequences of each Brassicaceae species using HMMER v3.2.1 software (https://github.com/Eddy RivasLab/hmmer/releases/tag/hmmer-3.2.1) with dome values less than 0.01 and the result output displayed as domtblout. All BRX family protein sequences were then searched via SynOrths (http://brassicadb.org/brad/tools/SynOrths/). Finally, all the BRX candidate proteins were confirmed by these two searches, resulting in a nonredundant protein list, and subjected to multiple sequence alignment using the ClustalW program with the default parameters. A phylogenetic tree was constructed using the maximum likelihood method with a bootstrap value of 1000 by MEGA 5. The online website iTOL (https://itol.embl.de/) was subsequently used to annotate the tree. The sequence length (SL), molecular weight (MW) and isoelectric point (IP) of the BRXL genes were obtained using tools from the ExPASy website (https://web.expasy.org/compute_pi/).

### Conserved domain, motif, and gene structure analysis

A location diagram of the conserved domain was constructed using TBtools software according to the relevant information from the NCBI Batch CD-search tool (https://www.ncbi.nlm.nih.gov/Structure/bwrpsb/bwrpsb.cgi). The conserved motifs were investigated using the MEME tool (http://meme-suite.org/tools/meme), with a number of found motifs of 10 and an optimum motif width of 6–200. The other parameters were set to the default values.

### Promoter cis-acting regulatory element predictions of *BRX* family genes

The promoter sequences (2 kb upstream of coding sequences) were identified, and promoter cis-element analysis was performed using the online analysis software associated with the PlantCARE database (http://bioinformatics.psb.ugent.be/webtools/plantcare/html/) and PLACE (https://www.dna.affrc.go.jp/PLACE/?action=newplace). The positions of the cis-elements were added to the promoter sequences using TBtools software.

### RNA-seq data and qRT-PCR analysis

To explore the expression patterns of *BrBRX* genes in different tissues of *B. rapa*, we adopted the Illumina RNA-seq data generated and analyzed by Tong et al. 41. Fragments per kilobase of exon per million mapped reads (FPKM) values were used to represent the gene expression levels. Heat maps of the gene FPKM values in *B. rapa* were generated using TBtools software.

To investigate the expression patterns of *BrBRX*s at different stages of heading and nonheading *B. rapa*, the Illumina RNA-seq data were analyzed, and the TPM (transcripts per million) or FPKM values were used to represent the gene expression levels. The expression patterns of *BrBRX* genes at the heading stage of Chinese cabbage are shown by different colors on the map.

For qRT-PCR analysis, total RNA was extracted from *B. rapa* leaves that had been treated with light/darkness or auxin. qRT-PCR was performed on an ABI QuantStudio 12K Flex Real-Time PCR system (Applied Biosystem, Foster City, CA, USA). The relative expression levels of specific genes were measured using the 2^−∆∆Ct^ method, and *BrActin3* was used as an internal control.

### Histochemical analysis of transgenic plants expressing Pro_*BrBRX*_:GUS fusion constructs

Three *BrBRX* promoter regions (~2 kb upstream of the three *BrBRX* genes) were amplified from Chiifu-401/42 and inserted into a pCAMBIA2300-GUS vector. The recombinant vectors were then transformed into Arabidopsis Col-0 via the floral-dip method. The histochemical localization of GUS was performed as described by Zhang et al.^18^.

### Generation and analysis of transgenic plants overexpressing *BrBRX*

The coding sequences of the three BrBRX genes were isolated from Chiifu-401/42 cDNA, after which they were cloned into pCAMBIA1300 binary vectors. The resulting constructs were subsequently transformed into Arabidopsis UK-1 (*brx*) and Col-0 plants via the floral-dip method. The transgenic lines were selected by screening seed progeny on MS media that were hygromycin resistant (supplemented with 50 mg/L) and then transplanted into soil. Because of frequent problems with transgene silencing in subsequent generations, the T2 generation was chosen for phenotypic analysis. A minimum of ten seedlings per line were investigated. To determine primary root length, the seedlings were grown on vertical plates and imaged with an SLR camera to generate an image file suitable for quantitative analysis via ImageJ software. The lengths of leaves, petioles, and seed pods were measured using a Vernier caliper. Leaf sections were obtained through paraffin embedding techniques and observed under an optical microscope.

## Supplementary information

Supplemental material 1

Supplemental material 2

## References

[1] Wang X (2011). The genome of the mesopolyploid crop species Brassica rapa. Nat. Genet..

[2] Liu S (2014). The Brassica oleracea genome reveals the asymmetrical evolution of polyploid genomes. Nat. Commun..

[3] Yang J (2016). The genome sequence of allopolyploid Brassica juncea and analysis of differential homoeolog gene expression influencing selection. Nat. Genet..

[4] Cui Y (2020). Segmental translocation contributed to the origin of the Brassica S-locus. Hortic. Plant J..

[5] Cheng F, Wu J, Wang X (2014). Genome triplication drove the diversification of Brassica plants. Hortic. Res..

[6] Cheng F (2016). Subgenome parallel selection is associated with morphotype diversification and convergent crop domestication in Brassica rapa and Brassica oleracea. Nat. Genet..

[7] Briggs GC, Mouchel CF, Hardtke CS (2006). Characterization of the plant-specific BREVIS RADIX gene family reveals limited genetic redundancy despite high sequence conservation. Plant Physiol..

[8] Beuchat J (2010). A hyperactive quantitative trait locus allele of Arabidopsis BRX contributes to natural variation in root growth vigor. Proc. Natl Acad. Sci. Usa..

[9] Mouchel CF, Briggs GC, Hardtke CS (2004). Natural genetic variation in Arabidopsis identifies BREVIS RADIX, a novel regulator of cell proliferation and elongation in the root. Genes Dev..

[10] Liu J, Liang D, Song Y, Xiong L (2010). Systematic identification and expression analysis of BREVIS RADIX-like homologous genes in rice. Plant Sci..

[11] Beuchat J (2010). BRX promotes Arabidopsis shoot growth. N. Phytol..

[12] Li Z (2019). OsBRXL4 regulates shoot gravitropism and rice tiller angle through affecting LAZY1 nuclear localization. Mol. Plant.

[13] Blanc G, Wolfe KH (2004). Functional divergence of duplicated genes formed by polyploidy during Arabidopsis evolution. Plant Cell.

[14] Casneuf T, De Bodt S, Raes J, Maere S, Van de Peer Y (2006). Nonrandom divergence of gene expression following gene and genome duplications in the flowering plant Arabidopsis thaliana. Genome Biol..

[15] Duarte JM (2006). Expression pattern shifts following duplication indicative of subfunctionalization and neofunctionalization in regulatory genes of Arabidopsis. Mol. Biol. Evol..

[16] Cheng F (2011). BRAD, the genetics and genomics database for Brassica plants. BMC Plant Biol..

[17] Bowers JE, Chapman BA, Rong J, Paterson AHJN (2003). Unravelling angiosperm genome evolution by phylogenetic analysis of chromosomal duplication events. Natture.

[18] Zhang J (2015). Three genes encoding AOP2, a protein involved in aliphatic glucosinolate biosynthesis, are differentially expressed in Brassica rapa. J. Exp. Bot..

[19] Tong C, Wang X, Yu J, Wu J, Liu SJBG (2013). Comprehensive analysis of RNA-seq data reveals the complexity of the transcriptome in Brassica rapa. BMC Genomics.

[20] Yamaguchi T, Nukazuka A, Tsukaya H (2012). Leaf adaxial-abaxial polarity specification and lamina outgrowth: evolution and development. Plant Cell Physiol..

[21] Wang Y, Wu F, Bai J, He Y (2014). BrpSPL9 (Brassica rapa ssp. pekinensis SPL9) controls the earliness of heading time in Chinese cabbage. Plant Biotechnol. J..

[22] Sun X (2019). Genome-wide transcriptome analysis reveals molecular pathways involved in leafy head formation of Chinese cabbage (Brassica rapa). Hortic. Res..

[23] He YK, Xue WX, Sun YD, Yu XH, Liu PL (2000). Leafy head formation of the progenies of transgenic plants of Chinese cabbage with exogenous auxin genes. Cell Res..

[24] Mao Y (2014). MicroRNA319a-targeted Brassica rapa ssp. pekinensis TCP genes modulate head shape in chinese cabbage by differential cell division arrest in leaf regions. Plant Physiol..

[25] Romano, C. P., Robson, P. R. H., Smith, H., Estelle, M. & Klee, H. Transgene-mediated auxin overproduction in *Arabidopsis*: hypocotyl elongation phenotype and interactions with the *hy6-1* hypocotyl elongation and *axr1* auxin-resistant mutants. *Plant Mol. Biol.* **27**, 1071–1083 (1995). 10.1007/BF00020881 7766890

[26] Kidner CA, Timmermans MC (2007). Mixing and matching pathways in leaf polarity. Curr. Opin. Plant Biol..

[27] Wu F (2007). The N-terminal double-stranded RNA binding domains of Arabidopsis HYPONASTIC LEAVES1 are sufficient for pre-microRNA processing. Plant Cell.

[28] Han MH, Goud S, Song L, Fedoroff N (2004). The Arabidopsis double-stranded RNA-binding protein HYL1 plays a role in microRNA-mediated gene regulation. Proc. Natl Acad. Sci. USA..

[29] Liu Z, Jia L, Wang H, He Y (2011). HYL1 regulates the balance between adaxial and abaxial identity for leaf flattening via miRNA-mediated pathways. J. Exp. Bot..

[30] Yu XH (2000). Cloning and structural and expressional characterization of BcpLH gene preferentially expressed in folding leaf of Chinese cabbage. Sci. China Ser. C.-Life Sci..

[31] Liang J, Liu B, Wu J, Cheng F, Wang X (2016). Genetic variation and divergence of genes involved in leaf adaxial-abaxial polarity establishment in Brassica rapa. Front. Plant Sci..

[32] Mouchel CF, Osmont KS, Hardtke CS (2006). BRX mediates feedback between brassinosteroid levels and auxin signalling in root growth. Nature.

[33] Scacchi E (2009). Dynamic, auxin-responsive plasma membrane-to-nucleus movement of Arabidopsis BRX. Development.

[34] Marhava P (2018). A molecular rheostat adjusts auxin flux to promote root protophloem differentiation. Nature.

[35] Scacchi E (2010). Spatio-temporal sequence of cross-regulatory events in root meristem growth. Proc. Natl Acad. Sci. USA.

[36] Rodrigues A (2009). The short-rooted phenotype of the brevis radix mutant partly reflects root abscisic acid hypersensitivity. Plant Physiol..

[37] Kliver, S. et al. Assembly of the Boechera retrofracta genome and evolutionary analysis of apomixis-associated genes. *Genes* **9**, 185 (2018). 10.3390/genes9040185 PMC5924527 29597328

[38] Gan, X. et al. The Cardamine hirsuta genome offers insight into the evolution of morphological diversity. *Nat. Plants* **2**, 16167 (2016). 10.1038/nplants.2016.167 PMC8826541 27797353

[39] Zhang T (2019). Genome of Crucihimalaya himalaica, a close relative of Arabidopsis, shows ecological adaptation to high altitude. Proc. Natl Acad. Sci. USA.

[40] Dorn KM, Fankhauser JD, Wyse DL, Marks MD (2015). A draft genome of field pennycress (Thlaspi arvense) provides tools for the domestication of a new winter biofuel crop. DNA Res..

