# Supplementary material for: Divergence of three BRX homoeologs in Brassica rapa and its effect on leaf morphology
Source: Hortic Res. 2021 Apr 1;8:68. doi: 10.1038/s41438-021-00504-3 (PMC8012600; doi:10.1038/s41438-021-00504-3)
Supplement: Supplementary file 1 — Supplemental material 1 [file 41438_2021_504_MOESM1_ESM.docx]

**Table S1** Sequence summary of the *BrBRX* genes indentified in the current study

| **Gene Name** | **Gene ID** | **CDS(bp）** | **Protein(aa)** | **No. of exons(size in bp)** | **No. of introns (size in bp)** |
| --- | --- | --- | --- | --- | --- |
| ***BrBRX.1*** | BraA09g033250.3C/ Bra023219 | 1026 | 341 | 5(79,317,130,392,108) | 4（899,223,118,138） |
| ***BrBRX.2*** | BraA08g009040.3C/ Bra035521 | 1014 | 337 | 5（79,314,130,383,108） | 4（2424,193,100,185） |
| ***BrBRX.3*** | BraA05g023900.3C/ Bra033869 | 975 | 324 | 4（336,130,395,114） | 3（448,146,77） |

**Table S2** Amino acid sequence identify (%) of BRX among *A. thaliana* and *B. rapa*

|  | ***AtBRX*** | ***BrBRX.1*** | ***BrBRX.2*** | ***BrBRX.3*** |
| --- | --- | --- | --- | --- |
| ***AtBRX*** | 100 | 93.4 | 89.88 | 88.82 |
| ***BrBRX.1*** |  | 100 | 91.69 | 90.97 |
| ***BrBRX.2*** |  |  | 100 | 87.74 |
| ***BrBRX.3*** |  |  |  | 100 |

**Table S3** Characteristics of the *BRX* family genes in *B.rapa*

| Gene _Name | Gene_ID | Chromosome |  |  | Stand | Extron | Intron | gene_Length | Protein_Length | PI | MW |
| --- | --- | --- | --- | --- | --- | --- | --- | --- | --- | --- | --- |
|  |  | No. | Start | End |  |  |  |  |  |  |  |
| *BrBRX.1* | Bra023219 | A09 | 20627199 | 20629602 | - | 5 | 4 | 2403 | 341 | 6.61 | 38676.77 |
| *BrBRX.2* | Bra035521 | A08 | 8148358 | 8152273 | - | 5 | 4 | 3915 | 337 | 6.23 | 38058.98 |
| *BrBRX.3* | Bra033869 | A05 | 15609292 | 15610937 | - | 4 | 3 | 1645 | 324 | 7.09 | 36217 |
| *BrBRXL1.1* | Bra005326 | A05 | 4848845 | 4851286 | + | 5 | 4 | 2442 | 335 | 5.84 | 38193.74 |
| *BrBRXL1.2* | Bra017311 | A04 | 15514622 | 15516749 | - | 5 | 4 | 2127 | 335 | 5.46 | 38078.68 |
| *BrBRXL2.1* | Bra027379 | A05 | 21332233 | 21334396 | + | 5 | 4 | 2164 | 362 | 5.51 | 40465.46 |
| *BrBRXL2.2* | Bra021525 | A01 | 23926678 | 23928513 | + | 5 | 4 | 1836 | 363 | 5.59 | 40659.95 |
| *BrBRXL3.1* | Bra037970 | A06 | 361100 | 363192 | - | 5 | 4 | 2092 | 364 | 5.27 | 40350.33 |
| *BrBRXL4.1* | Bra006563 | A03 | 4065426 | 4069134 | + | 5 | 4 | 3709 | 377 | 8.28 | 42081.8 |
| *BrBRXL4.2* | Bra020109 | A02 | 4050833 | 4054965 | + | 5 | 4 | 4133 | 372 | 7.61 | 41662.54 |

**Table S4** *BRX* family genes identified for phylogenetic analysis

**Table S5** The FPKM values of *BRXLs* genes in different tissues of *B. rapa*

| Gene_Name | Gene_Id | Flower | Leaf | Root | Silique | Stem |
| --- | --- | --- | --- | --- | --- | --- |
| *BrBRX.1* | Bra023219 | 3.30018 | 1.131945 | 6.074325 | 2.35026 | 1.80813 |
| *BrBRX.2* | Bra035521 | 1.06249 | 1.38755 | 0.7713915 | 0.274393 | 3.56277 |
| *BrBRX.3* | Bra033869 | 1.26284 | 0.396696 | 2.88027 | 0.380491 | 1.10157 |
| *BrBRXL1.1* | Bra005326 | 0.152687 | 0.5242815 | 5.34255 | 0.368035 | 1.5496 |
| *BrBRXL1.2* | Bra017311 | 0 | 0.1660846 | 0.2793658 | 0.0920086 | 0.0966343 |
| *BrBRXL2.1* | Bra027379 | 4.4519 | 0.8756355 | 4.85147 | 3.57693 | 3.58638 |
| *BrBRXL2.2* | Bra021525 | 2.81884 | 1.948775 | 2.254715 | 1.1041 | 4.20242 |
| *BrBRXL3* | Bra037970 | 2.81112 | 5.523105 | 7.892865 | 1.01638 | 18.8145 |
| *BrBRXL4.1* | Bra006563 | 6.51465 | 5.126765 | 42.81285 | 18.5653 | 11.3654 |
| *BrBRXL4.2* | Bra020109 | 0.962788 | 1.0134955 | 10.16513 | 1.74052 | 7.15497 |

**Table S6** Markers used for validating the selection signals of *BrBRX.1* and *BrBRX.2* gene in

*B. rapa* germplasm

| Gene | Mutation site | Genotype | Dominant in H-Br | Marker name | Primers |
| --- | --- | --- | --- | --- | --- |
| BrBRX.1 Bra023219 | 1698 | C/T | C | BrBRX.1_AlleleFAM | GAAGGTGACCAAGTTCATGCTGCTTGGCGGTTAAATTTCTGTACG |
|  |  |  |  | BrBRX.1_AlleleHEX | GAAGGTCGGAGTCAACGGATTGCTTGGCGGTTAAATTTCTGTACA |
|  |  |  |  | BrBRX.1_Common | TACTCGTGTCTTGTTTTGAAAGCC |
| BrBRX.2 Bra035521 | 1318 | T/C | T | BrBRX.2_AlleleFAM | GAAGGTGACCAAGTTCATGCTTGTCCTTTCTTCATGGGGCTG |
|  |  |  |  | BrBRX.2_AlleleHEX | GAAGGTCGGAGTCAACGGATTTGTCCTTTCTTCATGGGGCTA |
|  |  |  |  | BrBRX.2_Common | TCTGGTGCTTATAAACAATGCAAGCC |

**Table S7** Validation of the candidate genes in a *B rapa* germplasm collection with 908 accessions

| Genes | Group | Genotype A | Genotype B | P value |
| --- | --- | --- | --- | --- |
|  |  |  |  | (Fisher's exact test) |
| BrBRX.1 | H-Br | 160 | 159 | 8.43E-48 |
|  | NH-Br | 24 | 417 |  |
| BrBRX.2 | H-Br | 172 | 194 | 6.51E-49 |
|  | NH-Br | 22 | 438 |  |

**Table S8** The FPKM values of *BrBRX* genes in leafy head of Chinese cabbage

| GeneID | *BrBRX.1* | *BrBRX.2* | *BrBRX.3* |
| --- | --- | --- | --- |
| SA | 1.2239645 | 1.3949575 | 0.3804155 |
| HL1-1 | 3.8728655 | 2.8926745 | 1.2926265 |
| HL2-1 | 3.504271 | 3.6649635 | 1.4203925 |
| HL2-2 | 1.8805075 | 0.989946 | 0.3513685 |
| HL3-1 | 2.5158105 | 4.1590295 | 1.591772 |
| HL3-2 | 2.753131 | 3.46075 | 1.068323 |
| HL3-3 | 2.9636265 | 3.189902 | 1.010664 |
| HL3-4 | 3.008626 | 1.056084 | 0.3931355 |
| HL3-5 | 0.9833365 | 0.328775 | 0.078873 |
| HL5-1 | 4.870823 | 1.931291 | 0.746264 |
| HL5-2 | 4.404264 | 3.0154895 | 1.133635 |
| HL5-3 | 3.599755 | 2.762863 | 1.300343 |
| HL5-4 | 3.173965 | 1.460125 | 0.848834 |
| HL5-5 | 1.0449335 | 1.544289 | 0.1938015 |
| HL7-1 | 3.369151 | 6.5856265 | 1.048804 |
| HL7-2 | 4.417338 | 8.025907 | 0.90829 |
| HL7-3 | 3.99305 | 7.953867 | 0.793262 |
| HL7-4 | 3.430531 | 5.255736 | 1.184224 |
| HL7-5 | 2.403484 | 5.887007 | 0.9872005 |
| HL9-1 | 0.2313585 | 0.5823735 | 0.3792985 |
| HL9-2 | 0.546453 | 0.8824795 | 0.078046 |
| HL9-3 | 1.211024 | 1.0790215 | 0.42108 |
| HL9-4 | 1.9635775 | 4.5275055 | 0.270224 |
| HL9-5 | 2.082057 | 5.6758435 | 0.168761 |

**Table S9** Primer sequences used in this article.

| Primer Name | Primer sequence(5'-3') | purpose |
| --- | --- | --- |
| *BrBRX.1*-ES(F) | CCGGAATTCATGTTCACTTGCATAACTTGTACCAAAGC | Vector construction |
| *BrBRX.1*-ES(R) | TCCCCCGGGGAGGTATTGGCTTTGTATTCTCTCTCT |  |
| *BrBRX.2*-ES(F) | CCGGAATTCATGTTTACTTGCATAGCTTGTACGAAAGC |  |
| *BrBRX.2*-ES(R) | TCCCCCGGGGGAGGTATTGAGTTTGTATTCTCTCCCTGT |  |
| *BrBRX.3*-ES(F) | CCGGAATTCATGTTTACTTGCATAGCTTGTACGAAAGC |  |
| *BrBRX.3*-ES(R) | TCCCCCGGGGCTTAGAAGTTTGCTATACCTGAATCTGAC |  |
| *BrBRX.1*-Promoter-F | acgacggccagtgccaagcttTCGACATCGCACGAAGAAGAA | Promoter cloning |
| *BrBRX.1*-Promoter-R | ataagggactgaccacccgggCTTTGCCTCTTTTTGTGTTCGC |  |
| *BrBRX.2*-Promoter-F | acgacggccagtgccaagcttAGGTAGGTATTTATAGCCTCCAAAATAA |  |
| *BrBRX.2*-Promoter-R | ataagggactgaccacccgggTCTCTTTGCCTTTACAGCCCTATA |  |
| *BrBRX.3*-Promoter-F | gccaagcttgcatgcctgcagATCGATTCGTCACTCGTCTCTATTAT |  |
| *BrBRX.3*-Promoter-R | ataagggactgaccacccgggGGGATCCACAATCCGACATCA |  |
| *BrBRX.1*-qPCR-F | GTGGAAGCACCACTCCCAAT | Real-time quantitative PCR |
| *BrBRX.1*-qPCR-R | CTTCATGGGGCTGGTAGATGA |  |
| *BrBRX.2*-qPCR-F | TAGCCGGGAGACATTCGAC |  |
| *BrBRX.2*-qPCR-R | ACTGGTCATCGGATTTTGCC |  |
| *BrBRX.3*-qPCR-F | GTCCATATCACATTCTCGTCGC |  |
| *BrActing2-*F | GGAGCTGAGAGATTCCGTTG |  |
| *BrActing2-R* | GAACCACCACTGAGGACGAT |  |
| *BrBRX.3*-qPCR-R | GTTGAGCTTGCCACTTGTCG |  |
|  |  |  |


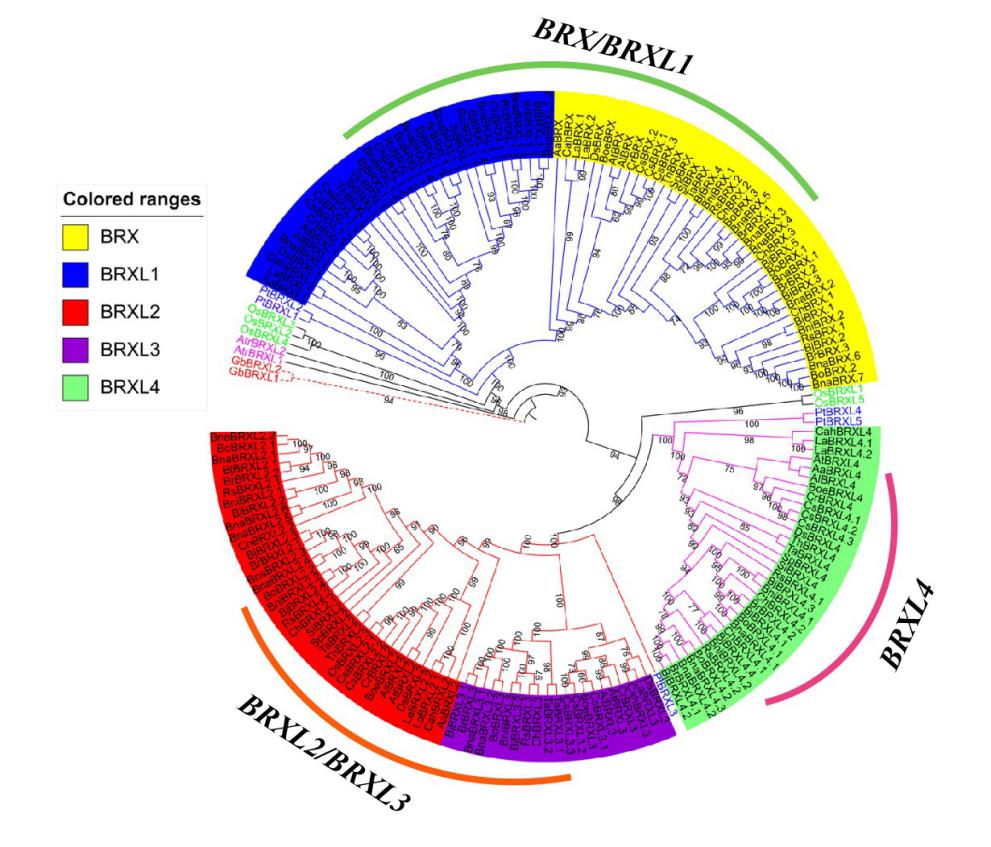


**Fig. S1 Phylogenetic analysis of *BRX* family genes from 21 species of Brassicaceae.** The phylogenic tree was constructed using the Maximum likelihood method with the bootstrap values of 1000 by MEGA 5. The red dashed line represents the outgroup sequence. The words of different colors represent ginkgo(red), Amborella trichopoda (pink), rice (green), and polar (blue). In the phylogenetic tree, BRX family genes were clustered into three clades.


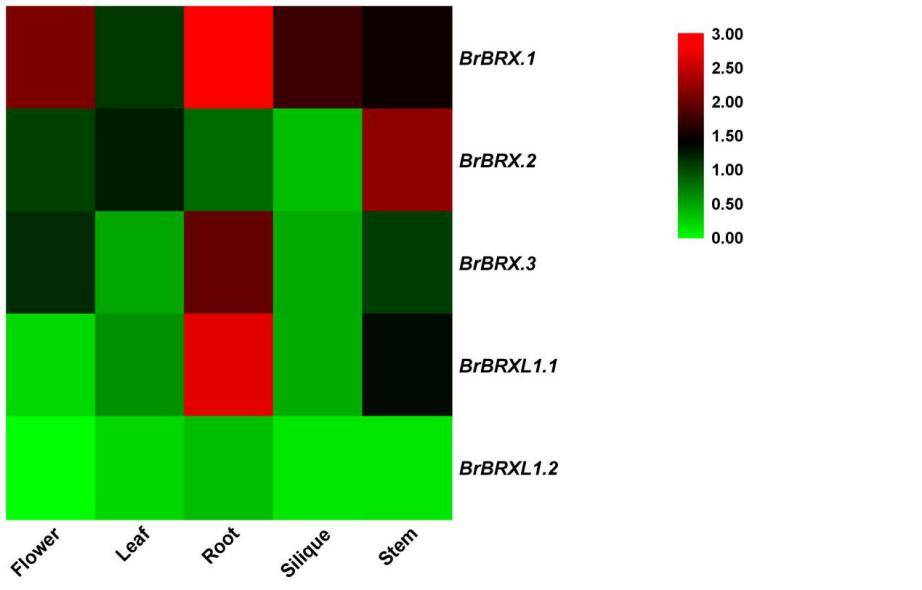


**Fig. S2** Expression of *BrBRX and BrBRXL1* in different tissues.


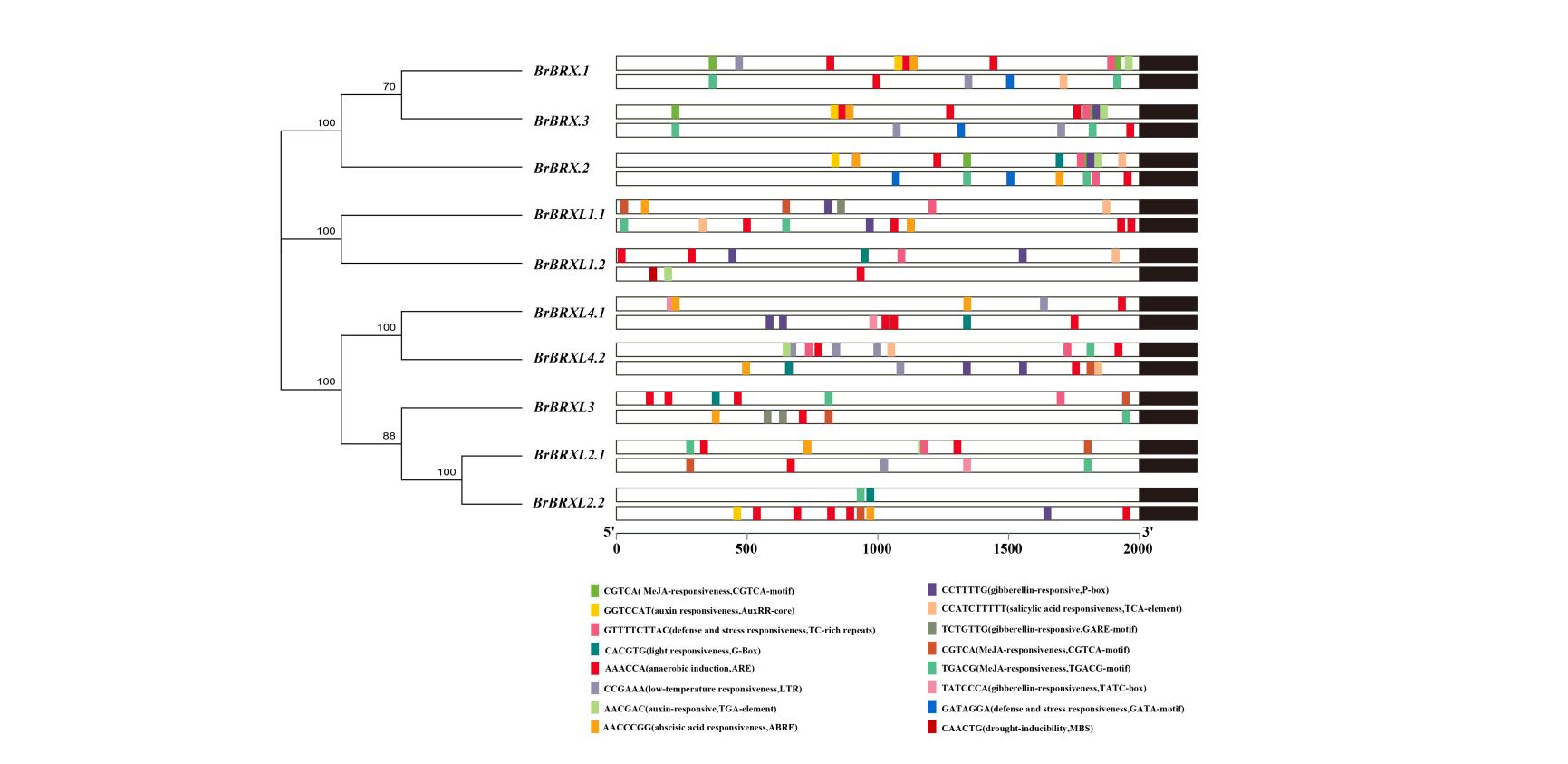


**Fig. S3** The promoter cis-elements analysis of *BrBRXLs*. The 2 kb DNA framents upstreamof the ATG staring code of *BrBRXs* were analyzed using online analysis software PlantCARE(http://bioinformatics.psb.ugent.be/webtools/plantcare/html/) and PLACE(<https://www.dna.affrc.go.jp/PLACE/?action=newplace>).


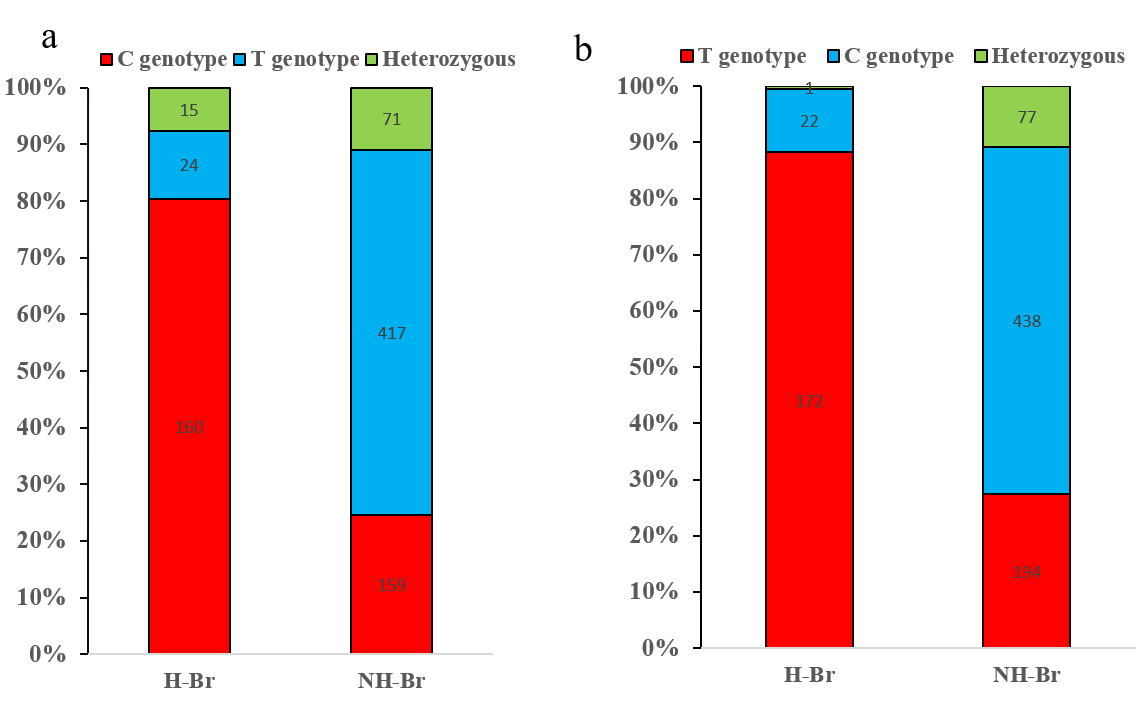


**Fig. S4 Analysis of selected sites of *BrBRX.1* and *BrBRX.2.*** In a larger *B. rapa* collection of

908 accessions, the C genotype in *BrBRX.1* (a) and the T genotype in *BrBRX.2* (b) were strongly associated with the leaf heading trait.


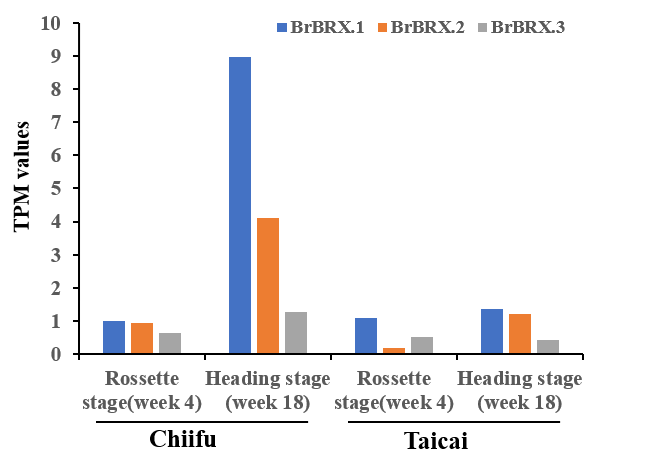


**Fig. S5** Transcript abundance profile of the three *BrBRX* genes at rosette stage (week 4) and heading stage (week 18) of heading accession ‘Chiifu’ and at the identical time points (week 4 and week 18) of non-heading accession ‘Taicai’.
