# Supplementary material for: Divergence of three BRX homoeologs in Brassica rapa and its effect on leaf morphology
Source: Hortic Res. 2021 Apr 1;8:68. doi: 10.1038/s41438-021-00504-3 (PMC8012600; doi:10.1038/s41438-021-00504-3)
Supplement: Supplementary file 2 — Supplemental material 2 [file 41438_2021_504_MOESM2_ESM.pdf]

Table S4 *BRX* family genes identified for phylogenetic analysis

Species	Gene Name	Gene ID	References
A.arabicum	*AaBRX*	AA_scaffold3949_23	Haudry et al., 2013
	AaBRXL1	AA_scaffold1161_27	
	AaBRXL2	AA_scaffold5648_33	
	AaBRXL4	AA_scaffold1068_224	
A.lyrata	*AlBRX*	fgenesh2_kg.1__3330	Benderoth et al., 2006
	AlBRXL1	scaffold_402165.1	
	AlBRXL2	fgenesh2_kg.3__1509	
	AlBRXL3	Al_scaffold_0001_4862	
	AlBRXL4	fgenesh2_kg.6__2092	
A.thaliana	*AtBRX*	AT1G31880	Kroymann et al., 2003
	AtBRXL1	AT2G35600	
	AtBRXL2	AT3G14000	
	AtBRXL3	AT1G54180	
A.trichopoda	*AtrBRXL1*	scaffold00024.190	Genome et al., 2013
	AtrBRXL2	scaffold00048.185	
	AtBRXL4	AT5G20540	
B.juncea	*BjBRX.1*	BjuA046002	Yang et al., 2016
	BjBRX.2	BjuA042760	
	BjBRX.3	BjuA028356	
	BjBRX.4	BjuB000575	
	BjBRX.5	BjuO010475	
	BjBRXL1.1	BjuA016973	
	BjBRXL1.2	BjuA018337	
	BjBRXL1.3	BjuB026691	
	BjBRXL1.4	BjuB020296	
	BjBRXL2.1	BjuA006071	
	BjBRXL2.2	BjuA045303	
	BjBRXL2.3	BjuA038912	
	BjBRXL2.4	BjuB023887	
	BjBRXL3.1	BjuA028593	
	BjBRXL3.2	BjuB029364	
	BjBRXL4.1	BjuA041512	
	BjBRXL4.2	BjuA009324	
	BjBRXL4.3	BjuB014356	
	BjBRXL4.4	BjuB015294	
B.napus	*BnaBRX.1*	GSBRNA2T00140097001	Chalhoub et al., 2014
	BnaBRX.2	GSBRNA2T00073616001	
	BnaBRX.3	GSBRNA2T00058577001	
	BnaBRX.4	GSBRNA2T00061327001	
	BnaBRX.5	GSBRNA2T00019185001	
	BnaBRX.6	GSBRNA2T00058575001	
	BnaBRX.7	GSBRNA2T00044386001	

	BnaBRXL1.1	GSBRNA2T00069681001	
	BnaBRXL1.2	GSBRNA2T00050321001	
	BnaBRXL1.3	GSBRNA2T00141243001	
	BnaBRXL1.4	GSBRNA2T00051482001	
	BnaBRXL2.1	GSBRNA2T00067242001	
	BnaBRXL2.2	GSBRNA2T00054022001	
	BnaBRXL2.3	GSBRNA2T00104746001	
	BnaBRXL2.4	GSBRNA2T00045616001	
	BnaBRXL2.5	GSBRNA2T00017957001	
	BnaBRXL2.6	GSBRNA2T00011633001	
	BnaBRXL3.1	GSBRNA2T00003326001	
	BnaBRXL3.2	GSBRNA2T00053726001	
	BnaBRXL4.1	GSBRNA2T00081362001	
	BnaBRXL4.2	GSBRNA2T00004324001	
	BnaBRXL4.3	GSBRNA2T00140839001	
B. nigra	*BniBRX.1*	BniB016334-PA	Yang et al., 2016
	BniBRX.2	BniB042913-PA	
	BniBRXL1.1	BniB010401-PA	
	BniBRXL1.2	BniB046189-PA	
	BniBRXL2.1	BniB034938-PA	
	BniBRXL2.2	BniB016644-PA	
	BniBRXL3	BniB006929-PA	
	BniBRXL4.1	BniB012806-PA	
	BniBRXL4.2	BniB026598-PA	
B. oleracea	*BoBRX.1*	Bol014042	Liu et al., 2014
	BoBRX.2	Bol002207	
	BoBRX.3	Bol005970	
	BoBRXL1.1	Bol037798	
	BoBRXL1.2	Bol000806	
	BoBRXL2.1	Bol005930	
	BoBRXL2.2	Bol040433	
	BoBRXL3.1	Bol039061	
	BoBRXL4.1	Bol036135	
	BoBRXL4.2	Bol034605	
B.retrofracta	*BoeBRX*	BOERETT00037896	Kliver et al., 2018
	BoeBRXL1	BOERETT00054123	
	BoeBRXL2	BOERETT00020271	
	BoeBRXL3	BOERETT00037418	
	BoeBRXL4	BOERETT00061093	
C.hirsuta	*CahBRX*	CARHR031220.1	Gan et al., 2016
	CahBRXL1	CARHR129780.1	
	CahBRXL2	CARHR088810.2	
	CahBRXL3	CARHR044530.1	
	CahBRXL4	CARHR192280.1	

C.himalaica	*ChBRX.1*	Crahi.0187s0011.1	Zhang et al., 2019
	ChBRX.2	Crahi.0140s0033.1	
	ChBRX.3	Crahi.2555s0001.1	
	ChBRXL1.1	Crahi.0439s0007.1	
	ChBRXL1.2	Crahi.0274s0007.1	
	ChBRXL2.1	Crahi.0385s0031.1	
	ChBRXL2.2	Crahi.1117s0021.1	
	ChBRXL3	Crahi.0136s0016.1	
	ChBRXL4.1	Crahi.0442s0003.1	
	ChBRXL4.2	Crahi.0235s0028.1	
C.sativa	*CsBRX.1*	Csa14g041860.1	Kagale et al., 2014
	CsBRX.2	Csa17g050150.1	
	CsBRX.3	Csa03g036270.1	
	CsBRXL1.1	Csa04g048940.1	
	CsBRXL1.2	Csa05g018660.1	
	CsBRXL1.3	Csa06g038100.1	
	CsBRXL2.1	Csa19g020590.5	
	CsBRXL2.2	Csa15g018270.2	
	CsBRXL2.3	Csa01g016480.1	
	CsBRXL3.1	Csa14g064820.1	
	CsBRXL3.2	Csa17g094700.1	
	CsBRXL3.3	Csa03g061500.1	
	CsBRXL4.1	Csa13g023440.1	
	CsBRXL4.2	Csa20g032470.1	
	CsBRXL4.3	Csa08g014340.1	
C.rubella	*CrBRX*	Carubv10009645m	Slotte et al., 2013
	CrBRXL1	Carubv10023756m	
	CrBRXL2	Carubv10013958m	
	CrBRXL3	Carubv10009614m	
	CrBRXL4	Carubv10000976m	
D.sophia	*DsBRX*	Desop.0214s0230.1	https://phytozome.jgi.doe .gov/pz/portal.html
	DsBRXL1	Desop.0009s0042.1	
	DsBRXL2	Desop.0227s0828.1	
	DsBRXL3	Desop.0079s0148.1	
	DsBRXL4	Desop.0240s0614.1	
G.biloba	*GbBRXL1*	Gb_13556	Guan et al., 2016
	GbBRXL2	Gb_18519	
L. alabamica	*LaBRX.1*	LA_scaffold2568_6	Haudry et al., 2013
	LaBRX.2	LA_scaffold2729_18	
	LaBRXL1	LA_scaffold2037_12	
	LaBRXL2.1	LA_scaffold1008_60	
	LaBRXL2.2	LA_scaffold801_70	
	LaBRXL3	LA_scaffold1976_45	

	LaBRXL4.1	LA_scaffold3006_3	
	LaBRXL4.2	LA_scaffold3295_1	
O.sativa	*OsBRXL1*	Os08g0462700	
	OsBRXL2	Os02g0700700	
	OsBRXL3	Os04g0600500	Goff et al., 2002
	OsBRXL4	Os03g0853500	
	OsBRXL5	Os12g0193000	
P.trichocarpa	*PtBRXL1*	fgenesh1_pm.C_scaffold_29000143	
	PtBRXL2	fgenesh1_pm.C_LG_III000279	
	PtBRXL3	estExt_fgenesh1_pg_v1.C_LG_III1601	Tuskan et al., 2006
	PtBRXL4	fgenesh1_pm.C_LG_VI000394	
	PtBRXL5	fgenesh1_pm.C_LG_XVIII000005	
R.sativus	*RsBRX.1*	Rsa10029942	Kitashiba et al., 2011
	RsBRX.2	Rsa10039586	
	RsBRXL1.1	Rsa10038760	
	RsBRXL1.2	Rsa10020526	
	RsBRXL2.1	Rsa10034783	
	RsBRXL2.2	Rsa10013098	
	RsBRXL3	Rsa10009183	
	RsBRXL4.1	Rsa10013165	
	RsBRXL4.2	Rsa10035832	
S.irio	*SiBRX*	SI_scaffold2037_38	Haudry et al., 2013
	SiBRXL1	SI_scaffold822_62	
	SiBRXL2	SI_scaffold2431_23	
	SiBRXL4	SI_C223667_1	
S.parvula	*SpBRX*	c0001_02461g	Dassanayake et al., 2011
	SpBRXL1	c0004_01197	
	SpBRXL2	c0005_01204	
	SpBRXL4	c0002_01046g	
T.arvense	*TaBRX*	Thlar.0053s0076.1	Dorn et al., 2015
	TaBRXL1	Thlar.0013s0478.1	
	TaBRXL2	Thlar.0016s0523.1	
	TaBRXL3.1	Thlar.0015s0108.1	
	TaBRXL3.2	Thlar.0015s0109.1	
	TaBRXL4	Thlar.0007s0187.1	
T. halophila	*ThBRX*	Thhalv10008095m	Yang et al., 2013a
	ThBRXL1	Thhalv10016906m	
	ThBRXL2	Thhalv10020939m	
	ThBRXL4	Thhalv10013806m	
